# Characterisation of Inactivation Domains and Evolutionary Strata in Human X Chromosome through Markov Segmentation

**DOI:** 10.1371/journal.pone.0007885

**Published:** 2009-11-25

**Authors:** Ashwin Kelkar, Vivek Thakur, Ramakrishna Ramaswamy, Deepti Deobagkar

**Affiliations:** 1 Department of Zoology, Pune University, Pune, India; 2 Centre for Computational Biology and Bioinformatics, School of Information Technology, Jawaharlal Nehru University, New Delhi, India; 3 Global Theme - Biotechnology, International Crops Research Institute for the Semi-Arid Tropics (ICRISAT), Patancheru, Andhra Pradesh, India; 4 School of Physical Sciences, Jawaharlal Nehru University, New Delhi, India; University of Pennsylvania School of Medicine, United States of America

## Abstract

Markov segmentation is a method of identifying compositionally different subsequences in a given symbolic sequence. We have applied this technique to the DNA sequence of the human X chromosome to analyze its compositional structure. The human X chromosome is known to have acquired DNA through distinct evolutionary events and is believed to be composed of five evolutionary strata. In addition, in female mammals all copies of X chromosome in excess of one are transcriptionally inactivated. The location of a gene is correlated with its ability to undergo inactivation, but correlations between evolutionary strata and inactivation domains are less clear. Our analysis provides an accurate estimate of the location of stratum boundaries and gives a high–resolution map of compositionally different regions on the X chromosome. This leads to the identification of a novel stratum, as well as segments wherein a group of genes either undergo inactivation or escape inactivation *in toto*. We identify oligomers that appear to be unique to inactivation domains alone.

## Introduction

In mammals, the X chromosome is present in two copies in the female and in one copy in the male. There is a unique evolutionary pressure on the X chromosome, as it spends 2/3 of the time inside female individuals. Since recombination between X and Y along the entire length is not possible, there is necessarily a divergence in the sequences of both these chromosomes [1]. The dosage of X linked genes is not identical in males and females, and a single copy of genes on the X chromosome in females is kept transcriptionally active so as to achieve equal dosage. The second copy of X chromosome is made inactive via transcriptional silencing [2].

**Figure 1:**
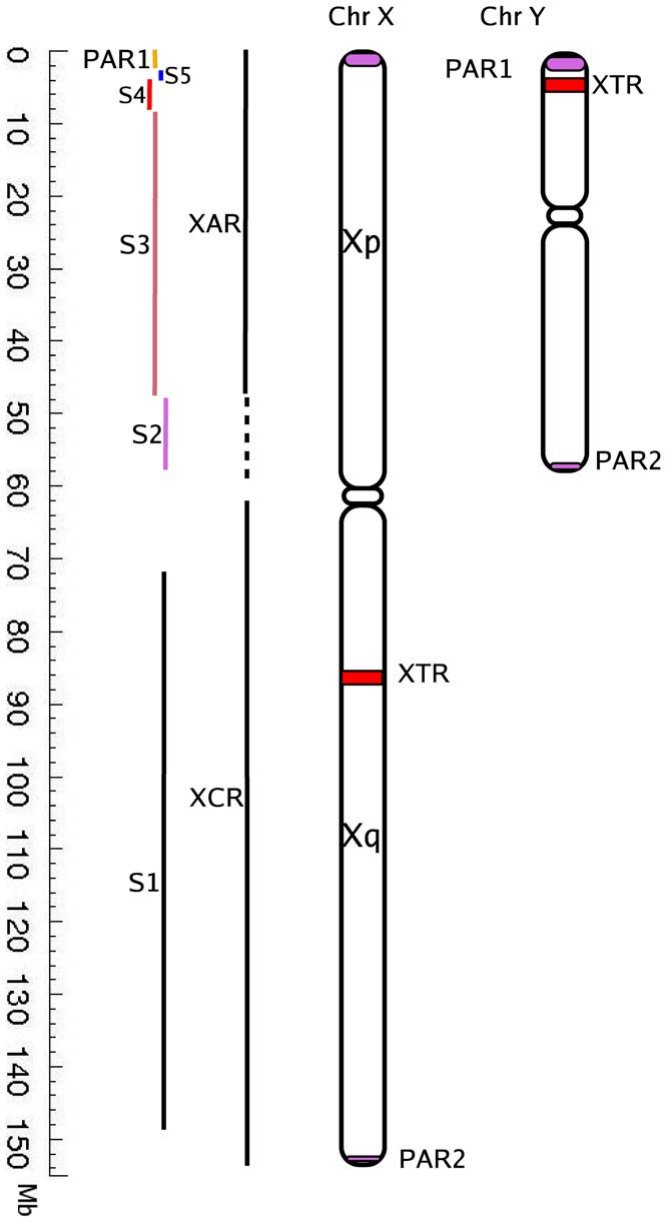
Schematic representation of human sex chromosomes. The evolutionary strata (S1–S5) as well as the conserved and acquired regions on the X-chromosome (XAR and XCR) are shown by vertical lines parallel to the chromosome. PAR1 and PAR2 are pseudoautosomal regions at both termini of the sex chromosomes and XTR is the X-transposed region. Chromosome Y has a homologous region to PAR1 on the X chromosome.

Figure 1 schematically depicts the organization of the human X chromosome. The human X chromosome has length 155 Mbp, with an overall GC content of 40% and a total of about 1500 genes, corresponding to a low gene density of less than 10 genes per Mb on average [4]. The data indicates that a major portion of the long arm, from Xqter to within a few Mbp of the centromere (the so-called XCR or X conserved region) has originated earlier than the major portion of the short arm of X, namely from Xpter to few Mbp of the centromere on the short arm which is known as the XAR or the X added region [4].

One of the enduring problems in the study of mammalian sex chromosomes has to do with their origin and function. They are believed to have evolved from a pair of autosomes and major evidence for this has come from sequence comparison of the human chromosome X with the complete genome of *G. gallus* [3], [4]. Sequence analysis and studies of synteny in the mammalian X chromosome suggests that there are several evolutionary strata [1], namely distinct portions of the chromosome that appear to have been acquired and/or originated at different evolutionary times and possibly from different organisms. There are five such evolutionary strata, S1 to S5, arranged in order from the distal end of the long arm to that of the short arm. S1 is the oldest stratum while S5 is the most recent. The current evolutionary landscape of the X chromosome is based largely on analysis of the substitution rate patterns in partial sequences of about 20 X-Y linked homologous genes spread over the chromosome length [1]. This has been further refined by comparison of complete DNA sequence with genomes of other species [4].

X chromosome structure plays an important role in its functional aspects: the location of a gene tends to decide its inactivation status [5], [6]. Inactivation occurs in *discrete domains* along X and all genes in a given domain tend to have a similar behaviour with respect to inactivation or escape from inactivation. Furthermore, genes in older strata such as the XCR tend to get inactivated, while genes on more recent strata (the XAR) tend to escape inactivation [1]. Studies on X- autosome translocations also indicate that the spread of inactivation onto the translocated X may or may not be complete and position effect variations have been reported [7]. Taken together, these observations suggest the presence of functionally distinct domains on the human X chromosome.

Regions of the genome that are functionally different often tend to have distinct compositions, for example the laterally transferred DNA against native sequences. Evolutionary events (such as rearrangements, selection pressure) are important factors which result in compositional variation along the sequence. This suggests that methods that can analyse and detect this compositional variation in a meaningful manner may prove useful in uncovering the origin of the functional variation as well. Earlier studies have shown that entropic segmentation is an effective technique for differentiating between regions of DNA that have distinct compositions [8], [9]. Segmentation involves the partitioning of a given DNA sequence into regions that are maximally distinct from one another based on a chosen compositional measure [11]. A commonly applied entropic segmentation strategy maximizes the Jensen-Shannon (JS) divergence [10] in order to construct this partition. In an earlier work we have shown that a *second* order Markov model, compared to non-Markovian and first-order models, is able to utilise nucleotide correlations and suffices in accurately partitioning portions of DNA in a statistically significant manner [12]. This technique accounts for trinucleotide correlations, and adequately distinguishes between genomic regions originating from different evolutionary events, such as genome islands versus native, duplications, etc [12].

In the present work we apply this Markov model based segmentation method [12] to the human X chromosome genomic sequence. As described above, the X chromosome is comprised of distinct evolutionary strata, the boundaries of which have been characterized largely on the basis of substitution rates of select X-Y gene homologues. Segmentation, however, can characterize structures at a much finer resolution. We show here that the domains obtained through segmentation have a close correspondence with the evolutionary strata that have been proposed for the X chromosome. Examination of the domain boundaries obtained via segmentation provides new insight into the distribution of genes that undergo or escape inactivation. Our data predicts a new boundary within the evolutionary stratum S3. This boundary separates the S3 into two regions that have distinct nucleotide composition, and also have differential inactivation status.

Another longstanding issue in the context of X inactivation is whether the signals that cause gene silencing are locally situated in the vicinity of genes or whether there is a chromosome wide signal that is used as a target by the inactivation machinery. Earlier studies that focused on the composition of regions where genes either undergo inactivation or escape inactivation have suggested a correlation between local sequence features and inactivation status [13]. We apply the pattern detection program TEIRESIAS [14] and identify a unique set of oligomers that occur in segments where all genes escape inactivation, and similarly, unique oligomers that occur in domains where genes do not escape inactivation. The distribution of such unique oligomers may provide clues to the distribution of signals that cause inactivation.

## Results

### Markov Segmentation of the X Chromosome

**Figure 2:**
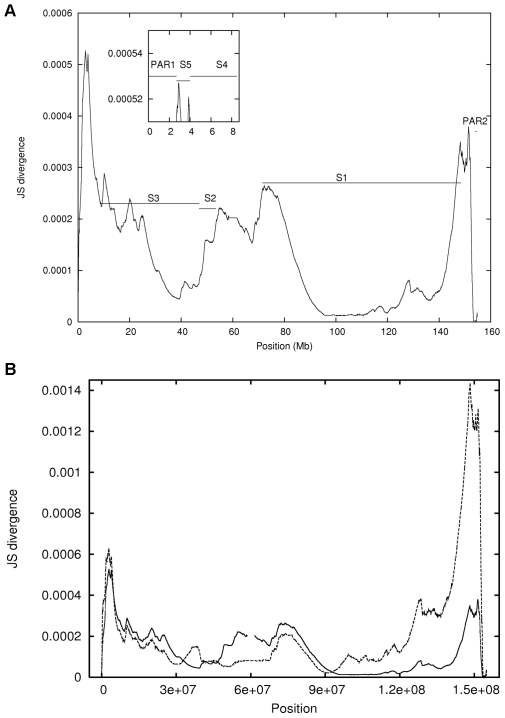
JS divergence as a function of segmentation position for (A) the unmasked X chromosome. The inset is a blow-up of the plot within the region of the highest (JS divergence) peak. (B) When the sequence is masked for repeats the overall shape of the JS divergence plot remains the same except for a change in the order of prominent peaks.

A plot of the JS divergence along the length of the sequence, as shown in Figure 2A, provides a visual representation of the compositional variability within human X chromosome. The global maximum occurs at the point where the sequence is *first* segmented (2.88 Mb), as the segmentation is a recursive process. Other prominent maxima (located at, for instance, 10.11 Mb, 72.13 Mb, and 151.5 Mb) correspond to the locations of subsequent segmentation points since the segmentation algorithm is applied recursively. The two most prominent maxima at 2.88 Mb and at 151.5 Mb indicate significant compositional differences between the terminal parts (proximal and distal ends) of the chromosome X and the rest of the chromosome. It is important to note that these terminal parts correspond very well with the two pseudo-autosomal regions (PAR1 and PAR2) (which still exchange DNA with their Y chromosome counterparts) and the variability pattern corroborates the distinctiveness now in terms of composition. Since repeats in any DNA sequence also contribute to variation in sequence composition, this analysis was repeated using the X chromosomal sequence masked for repeats using RepeatMasker [15]). The overall nature of plot is retained except that the order of the two prominent peaks located at 2.88 Mb and 151.5 Mb (Figure 2B) is reversed. This indicates that chromosome level organisation has been shaped by events which are largely insensitive to repeat distribution.

### Analysis of X Chromosomal Segmentation

**Figure 3:**
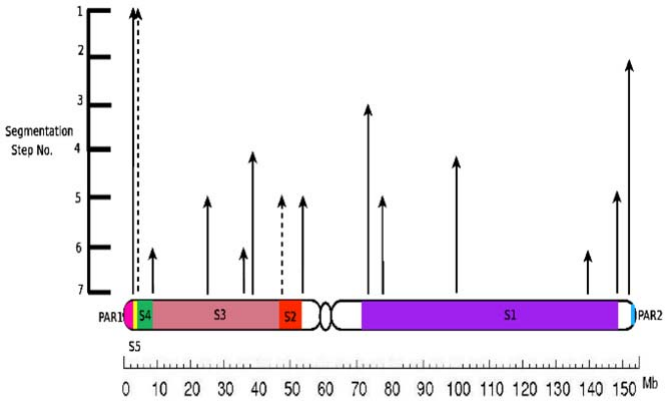
Segmentation boundaries in the unmasked X chromosome. The boundaries were obtained by second order Markov segmentation of the unmasked X chromosome up to the sixth step of recursive segmentation. The height of the corresponding arrows indicates the step number of segmentation. Some segmentation steps had more than one JS divergence maximum and these are indicated by the dashed arrows. (See Table S1 for the specific coordinates.).

We have carried out recursive segmentation until the first six steps which captures the compositional structures present in the chromosome. These segmentation points (Figure 3 and Table S1) correspond closely to the stratum boundaries that have been proposed from other studies: all but one stratum boundary lies within a distance of 2 Mb from a segmentation boundary predicted by our algorithm (Table 1).

**Table 1:** Boundaries of X-chromosome features showing correspondence with segmentation boundaries obtained from the first *six* steps (see Methods).

Feature	Reported Location (Mb)	Step no.	Nearest segmentation boundary (Mb)	Difference (Mb)
PAR1/S5	2.7	I	2.88	0.18
S4/S5	4	I	3.86̂	0.14
S3/S4	8.5	VI	9.04	0.54
S2/S3, XAR/XCR	47	V	49.3̂	2.3
S2 end	53.5	V	54.6	1.1
S1 start	71.5	III	73.8	2.3
S1 end	148.5	V	148.5	0

The boundaries labelled with ‘ ^’ indicate cases when the JS divergence in a segment had more than one maximum of equivalent heights. The age of the strata on the X chromosome in the order of older to newer is S1, S2, S3, S4, S5, PAR. These strata are arranged linearly on the X chromosome in order from oldest to newest. Thus the boundary between PAR1 and S5 is most recent while the one between S1 and the rest of the chromosome is oldest; see Figure 1.

The step at which the segmentation boundaries appears correlates with the age of the strata, and the regions of X chromosome which still exchange genetic material were identified at earlier steps of segmentation ( boundaries at 2.88 Mb at step I and 151.5 Mb at step 2) (Table S1). These regions are the most recently acquired by the X chromosome and therefore would be compositionally the most distinct from the rest of the X chromosome.

The segmentation boundaries that emerge at the third recursive step of segmentation coincide with the XAR/XCR boundary and the S1 boundary (Table S1). The XAR and XCR are known to have originated from two distinct autosomal sources at different time points [4] and therefore heterogeneity between these two strata is to be anticipated. Although XCR and XAR inclusion constitute the oldest events in the history of evolution of the X chromosome, compositional differences between them still persist (see Figure 3). In the fourth step, two prominent boundaries were observed (Figure S1) – one at ∼38 Mb and another at ∼99 Mb.These JS maxima correspond to boundaries between compositionally different sequences in these regions on the X chromosome. These two locations do not correlate to a known boundary of the X chromosomal evolutionary strata, and the compositional differences between the sequences on either side of these boundaries might not be a result of a different evolutionary origin.

### Compositional Structures within the XAR and Their Evolutionary/Functional Implications

**Figure 4:**
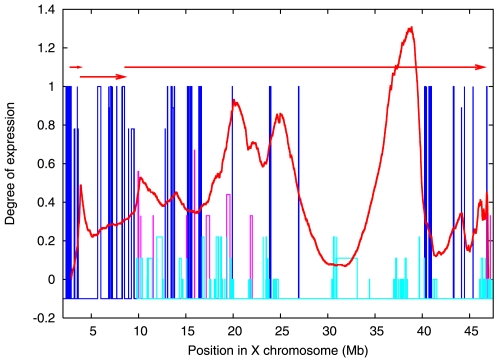
JS divergence values of unmasked XAR region (excluding PAR1). These divergence values were obtained using 2nd order Markov segmentation. XAR is comprised of three evolutionary strata (shown by the horizontal arrows). The additional structures that give rise to the different peaks are discussed in the text. The coloured bars represent normalized expression levels (number of clones showing expression divided by number of clones examined) of XAR genes examined in [6]. Blue boxes indicate transcriptionally active genes with expression level ranging between 0.75–1, while cyan boxes indicate inactivated genes with expression level <0.25. Expression levels of few genes (pink boxes) were intermediate of the two ranges and were considered ambiguous. The width of boxes represents the physical location of the gene.

Here we examine the XAR in details (see Figure 4). Two new boundaries are observed at the fourth step of segmentation: one boundary at (∼38 Mb) lies within XAR while the other lies in the middle of S1. XAR is well characterised and is known to contain three strata and one pseudo-autosomal region, PAR1. The PAR1 region exhibits homology with the terminal region of Y over a length of 10 Mb, but homologous genes in this region are largely non-syntenic. Availability of richer sequence information for XAR makes it suitable for further analysis and validation.

Shown in Figure 4 is the JS divergence along the XAR wherein at least 5–6 divergence peaks (local maxima) are visible. Two of these lie in the vicinity of two stratum boundaries S3/S4 and S4/S5, the last of which is a relatively recent report [4]. The boundaries identified by segmentation differ slightly from those reported in literature: we find PAR1-S5 boundary at 2.894 Mb, S5-S4 at 3.867 Mb and S4-S3 at 10.215 Mb. We also observe three new boundaries, all located within stratum S3.

The peak with highest JS divergence within S3 suggests the existence of distinct structures or domains within this stratum. This new divergence peak could be due to S3 itself being comprised of fragments with different origin. Comparative studies [4] of human XAR and the *G. gallus* autosome 1q, which shows occurrence of high degree of synteny suggests that this is improbable as the XAR is thought to have been derived from the same region of the *G.gallus* chromosome 1q and is thought to have been acquired by the mammalian X chromosome in a single evolutionary event.

**Figure 5:**
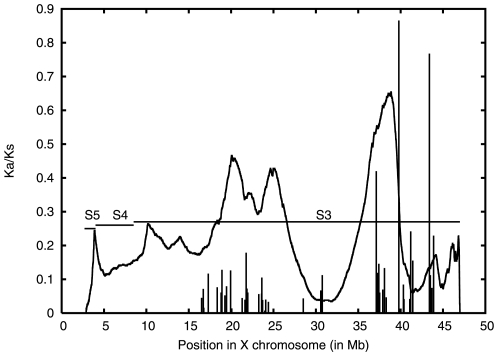
Ratio of synonymous (Ks) and non-synonymous (Ka) substitution rates of X-chromosome orthologs in the XAR. These rates were obtained from the pair of orthologs from *G. gallus* and human XAR, aligned by CLUSTALW2 [18], and analysed using the program DnaSP [19] (http://www.ub.es/dnasp/). The JS divergence (arbitrary scale) is superimposed on the plot.

If S3 has originated in single event, then the difference (between segments on either side of predicted boundary) could have been accumulated *after* these sequences came to reside on the X chromosome. To determine whether there is a differential selection pressure on the two regions, the rate of synonymous (Ks) and non synonymous (Ka) substitutions of the gene sequences of S3 with those of their syntenic homologues in *G. gallus* (in Chromosome 1) was analysed. About 50 pairs of homologous genes were identified based on pair wise comparison of protein sequences using the program *bl2seq* [16] (run at a stringent threshold E-value of 6 · 10^−13^). Genes between 38–47 Mb showed a significantly higher Ka/Ks ratio as compared to the genes from 15–31 Mb. ( There were no genes in the region 31–37 Mb that were suitable for analysis.) Figure 5 shows the Ka/Ks values plotted over the location of the genes (see also Table S2). A higher Ka/Ks ratio implies that more non-synonymous substitutions were allowed in these sequences during evolution. The significance of difference in the Ka/Ks values was inferred using Wilcoxon rank sum test meant for two independent samples with significance at 0.01 level. The alternate hypothesis states that the substitution ratios in the region 37–47 Mb are significantly greater than those from 15–31 Mb. Rejection of the null hypothesis confirmed relative sequence conservation of genes in the region 15–31 Mb than those lying in 37–47 Mb (The Z value computed for the sum of ranks for the smaller sample was 2.63, which was greater than the table value of Z (i.e. 2.33) at a significance level of 0.01).

One of the segments of S3, which was relatively conserved, was comprised of genes showing propensity to undergo inactivation. The expression profile derived by Carrel et al. [6] consists of measuring expression out of a total of 9 clones for each gene. The expression profile data from that experiment was considered for our analysis. The expression level was normalised using the number of clones for each gene and the number of clones showing positive expression for each gene. Out of 100 genes in the region 8.5 to 38 Mb, 91 genes show an expression pattern of being either completely exempt from inactivation ((norm. expression level > = 0.77)) or being completely inactivated (norm. expression level > = 0.22).Out of these 91, two-thirds (namely 60) of the genes showed poor expression while only 31 were found to be transcriptionally active. On the other hand, in the second segment (i.e. 38 to 64 Mb), genes with normalised expression < = 0.22 or > = 0.77 were in equal number (Table S3) (out of 28, 14 belonged to each category) [6]. Thus a larger fraction of genes undergoes inactivation on one side the JS divergence peak identified by the segmentation method, as compared to the other.

The present segmentation analysis therefore predicts an additional compositional boundary within what was reported to be a single evolutionary stratum on the X chromosome. This boundary also separates the two sub sequences into *functionally* distinct regions on the X chromosome with respect to the inactivation of the genes in those regions. The conservation of some of the genes on either side of the boundary also differs to a significant degree indicating that the changes must have been acquired after the genes came to reside on the X chromosome. Since the only difference in the subsequences on either side of the boundary, after being acquired by the X chromosome, could have been differential propensity of inactivation, these three observations suggest that compositional changes are correlated with the differential propensity of inactivation of the genes on either side of the boundary.

### Domains with Distinct Transcriptional Profile Display Different Compositional Makeup

About 15% of the genes on the inactive X chromosome are not silenced in the process of X inactivation. Whether a gene has propensity to be inactive or not partly depends on which stratum it resides in [5], [6]: it was previously observed that genes on older strata tend to get inactivated, while those on newer strata tend to escape inactivation. For instance, almost all genes in PAR (the most recent addition to the X chromosome) are expressed, while almost all the genes in XCR or S1 undergo inactivation. However most strata are constituted of domains of both types (one that undergoes inactivation while another which escapes).

Is this behaviour related to any compositional signal which differs between these distinct domains? An earlier study [13] has found that the 100 Kb upstream region of the two distinct classes of genes show difference in frequencies of certain repeats. This work, however, did not examine the basis of occurrence of such transcriptionally inactive/active domains. We analyse the domains in view of the previously determined inactivation status of genes [6]. To assess the ability of Markov segmentation to detect activation/inactivation boundaries, we analyzed a number of known domain pairs, namely sequences of DNA each comprised of a set of contiguous genes (equivalent to one known domain) known to undergo complete inactivation, lying adjacent to another such domain that entirely escapes inactivation, or *vice versa*.

**Figure 6:**
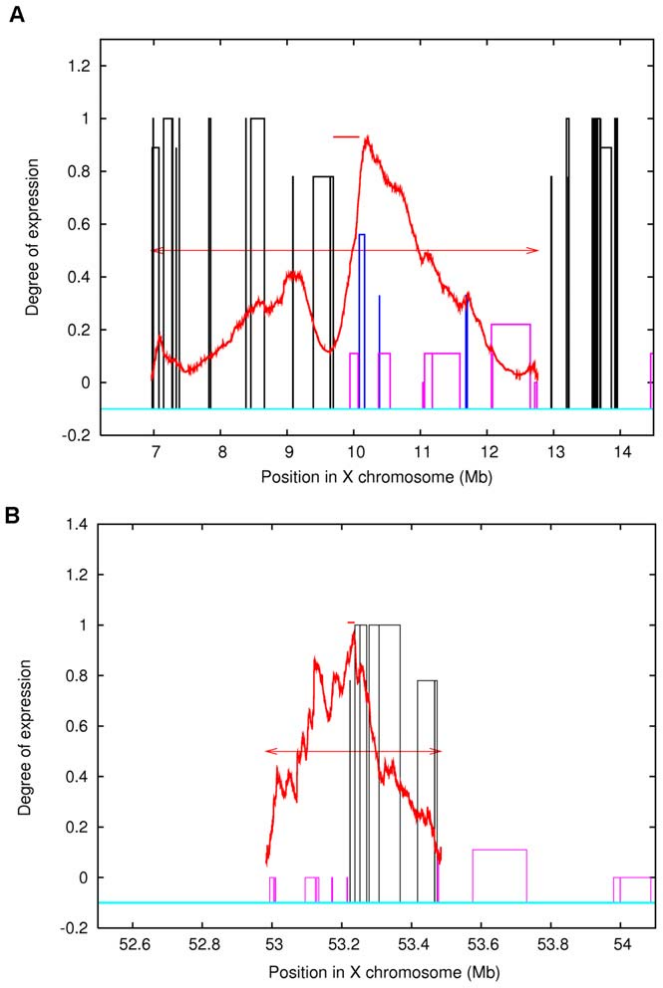
JS divergence plots of domain pairs. Figures A and B display the compositional variation in two sample domain pairs 1 & IX listed in Table 2. The arrows show the location of domains in the pair. The vertical bars are the normalised gene expression of the genes in these regions. The segmentation boundaries coincide with regions having differential gene expression. The divergence maximum occurs near a known boundary are suggestive of high compositional differences between these domains.

These sequences were subject to second–order Markov segmentation (see methods). The domain pairs were obtained using the data of Carrel *et al.* [6]. A total of 11 domain-pairs were segmented at the first step to obtain the first partition point (Table 2 and Table S4). The difference in location between the first partition point and the nearest domain boundary was considered the offset in distinguishing two domains. The offset was further normalized by dividing it by the size of the domain pair. The normalized values ranged between 0 and 0.53. For the domain pairs, where the predicted boundary was (empirically) reasonably close to the existing one, the maximum offset observed was about 0.12. (Figure 6, and Figure S2).

**Table 2:** Markov segmentation of domain pairs at the first partition point.

Domain	Strata	Start (Mb)	Size (Mb)	Normalized difference
I	S4	6.967	5.795	0.02
II	S3	10.075	3.884	0.03
III	S3	14.447	1.321	0.08
IV	S3	16.506	1.234	0.02
V	S3	23.585	0.566	0
VI	S3	40.358	1.319	0.01
VII	S3	46.182	0.803	0.08
VIII	S2	46.938	0.820	0.52
IX	S2	52.983	0.502	0
X	S2	53.227	1.184	0.12
XI	S1	72.947	1.124	0.39

Normalized difference is the distance between partition point and the nearest domain boundary, normalized by the domain-pair size.

This observation is suggestive of compositional differences playing an important role in determining the transcriptional properties of the genes on the X chromosome. If an entire domain of one kind (i.e. either escaping inactivation or undergoing inactivation) is compositionally uniform then the JS maxima will occur at the edges of each inactivation domain as is the case with 9 out of 11 pairs tested in this work. For the domain pairs where the JS maxima do not coincide with the existing predicted boundary or there are additional JS maxima within the domains we conclude that these might be due to highly skewed local composition and this may not have a direct bearing on inactivation status. Segmentation data is thus consistent with the fact that sequence information needed to predict inactivation status may be localized in clusters along the X chromosome rather than present dispersed throughout the sequence in a uniform manner. As inactivation is considered to occur in domains, it is a plausible assumption that the regulation of phenomena required for inactivation also occurs in a domain like fashion.

### Identification of Domain Specific Oligonucleotides

The domains obtained from Markov segmentation were distinctive with regard to both nucleotide composition as well as gene inactivation status. Compositional difference between the segments is attributed to the skewed distribution (including exclusive presence or absence) of nucleotides or oligonucleotides of variable length. There have been several attempts to understand the role of sequence based factors in gene inactivation or escape from inactivation in X_i_; the involvement of repeats, for example, has been studied in some details and *causal association* between distribution of LINE-1 elements in gene neighbourhoods and inactivation status has been reported [17], [18]. A related study [13] examined the repeat features in 100 Kb upstream of the two classes of genes and reported, in addition to L1, the role of Alu and short motifs. These investigations however don't take into account the domain organisation, based on transcriptional patterns, of X chromosome. In order to identify compositional elements that are potentially involved in effecting inactivation (or escape), the program TEIRESIAS [14] was used to identify patterns in the DNA sequences that are *unique* to one type of dataset (the input sequence used was masked for low complexity repeats).

We consider eighteen domains wherein all genes undergo inactivation, and seven domains wherein all genes escape inactivation as the input data set (Table S5) to TEIRESIAS to detect patterns of minimal length 9 bp (see methods). Although a large observed in each set, we used program features within TEIRESIAS for removing redundancy and focused only on patterns that were unique to either set (those differentially distributed between the two sets were excluded). Among the non-redundant set of core patterns we identified 7 that occur exclusively in domains that escape inactivation and 3 that occur exclusively in domains that undergo inactivation, although some of the patterns (of each set) shared a smaller motif (Table 3).

**Table 3:** List of domain specific patterns.

Total occurrences	No. of sequences showing pattern	Pattern	Length
Set I:Domain undergoing inactivation			
133	18	AGTA**GCTGGGA**TTACAGGC	19
359	18	AAAG**TGCTGGGA**TTACAG	18
404	18	CCAAAGT**GCTGGGA**	14
Set II:Domain escaping inactivation			
45	7	CTCAGCCTC**CTGAGTAGCTGGGA**	23
24	7	**CTGAGTAGCTGGGA** CTA	17
25	7	AAAATACAAAA**AATTAGCC**	19
33	7	**AATTAGCC** AGGCGT	14
42	7	GTTGCAGTGAGCCAAGAT	18
48	7	GCCTGGGTGACAGAGC	16
38	7	GATCATGCCACTGCA	15

Multiple alignment of all patterns from the first set show some redundancy which might reflect in their mapping to same locations in the sequence.

Since the same set of oligonucleotides have been found in all domains that undergo complete inactivation, and since the domains are from different evolutionary strata, these patterns could be correlated to inactivation (or escape from inactivation) rather than to the evolutionary strata. We further explored the functional aspects of these patterns: a) if their location display any association with the coding region or regulatory sequences, b) if all or few of them are among the known repetitive elements.

The larger sequences in each domain were found not to correlate with gene locations or transcription start sites. It was also not found in any of the repeat elements that are present in the human genome. This was confirmed using REPBASE ( http://www.girinst.org/censor/index.php ). The fact that a common sequence motif was found in all domains undergoing inactivation is indicative of this sequence being a probable part of the targeting mechanism of the inactivation machinery that is responsible for epigenetic modifications that are the requirement of inactivation.

## Materials and Methods

### X Chromosomal Sequence

Build NCBI36 of the human X-chromosome from ENSEMBL [19] is the input sequence for our analysis. The annotation used is specified at http://www.ncbi.nlm.nih.gov/Gene.

### Markov Segmentation

Entropic segmentation of a given DNA sequence proceeds as follows. The sequence S is partitioned into subsequences *S_1_* and *S_2_* so as to maximize the Jensen–Shannon (JS) divergence, namely the quantity
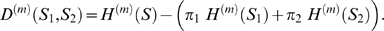
(1)


Here *H^(m)^* is the Shannon entropy, π's are weights of individual subsequences 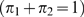 and the superscript *m* refers to the order of the Markov model that is used to describe the sequence.This is the simplest case of 1∶2 partitioning; more complicated partitions are possible.

The JS divergence is a measure of the difference between two distributions. In the present case, it computes the difference in nucleotide/oligonucleotide composition of subsequences under consideration, as quantified by the Shannon entropy. The Shannon entropy itself is a measure of the information contained in the sequence. In the (zeroth) 0^th^ order segmentation, the distribution of the four nucleotides (A, T, C, and G) is analysed, whereas in 1^st^ order the distribution of the sixteen dinucleotides, and in 2^nd^ order, the distribution of sixty-four trinucleotides are considered in the calculation of compositional differences. Since the increase in order of the Markov model implies that a more stringent sequence property is used to calculate compositional differences, a higher order model will in general give a more accurate representation of the sequence than a lower order one. While the zeroth order case has been most extensively studied earlier, it has been shown that a second order model is more appropriate for capturing the complexity of biological sequences as it is more accurate in prediction of boundaries between compositionally differing regions [12].

Operationally segmentation is carried out as follows. For a specific order, the divergence is computed for *all possible* partitions i.e. at each base along the sequence, ensuring that both subsequences *S_1_* and *S_2_* are larger than a specified threshold size. The sequence *S* is then segmented at the point of *maximum*, *D_max_^(m)^* if this value satisfies statistical significance criteria [10]. The current analysis was carried out at confidence level 0.99. The procedure is carried out recursively, namely *S_1_* and *S_2_* each are then further segmented, and each of the resulting segments are further segmented, and so on, until halting criteria are met. In the present work, the halting criteria that are used are that either *D_max_^(m)^* fails to have statistical significance or the segment size becomes smaller than the specified threshold [10]. Thus, a set of segments are obtained in a hierarchical manner.

In order to obtain a global pattern of compositional variation along a sequence, peak(s) in a plot of *D^(m)^* indicate location(s) of high heterogeneity. A prominent peak(s) will indicate position(s) having large compositional differences between segments to the left and right. More than one maximum can have similar *D^(m)^* values, and in such cases either of them can be considered for further segmentation.

Since the method is contextual in nature, note that all segmentation points (namely, those obtained until the final recursive segmentation step) may not necessarily correspond to peaks in a plot of *D^(m)^* of the original sequence. Moreover, in order to capture the major locations of heterogeneity and to identify large scale structures in X-chromosome we carry out segmentation only for a limited number of hierarchical steps. Small segments (of size less than 1 Mb, say) compared to the complete chromosome (∼150 Mb), were omitted.

### Teiresias

This program identifies oligonucleotides that occur within a sequence or set of sequences with a frequency higher than expected by chance. We briefly describe the manner in which this program was used in the present work. It is necessary to specify support, namely the number of occurrences of any pattern. The program then identifies the longest unique subsequences that occur at least as frequently as this specified number of times in the input sequence(s). Each input data is randomized to determine whether the identified subsequences are significant or not.

A standalone version of TEIRESIAS was used, with default parameters unless specified. Multiple instances of each of the two types of domains (X chromosomal sequences comprising of genes that either escape inactivation or undergo inactivation) were extracted (based on Carrel and Willard's data [6]). The s*upport* for a given set, namely inactive and active domains, was specified as the number of sequences present in each set and patterns with threshold length 9 and 10 were identified. Input sequences were masked for low complexity regions but repeat elements were not specifically excluded. The patterns obtained showed high redundancy and only the representative patterns were finally chosen based on a TEIRESIAS program for filtering redundant patterns.

## Discussion

In this paper we have applied a Markov model of genome segmentation in order to study the inherent correlations within the human X chromosomal DNA sequence. This method examines the base composition and higher order nucleotide correlations within the sequence and identifies domains wherein the intra-domain structure is homogeneous while adjacent domains are distinct. The Jensen–Shannon divergence provides a quantitative measure for the heterogeneity of the domains, and we show here that by using criteria based on this measure the segmentation procedure accurately probes the evolutionary structure of the X chromosome.

The mammalian X chromosome is unique in that it undergoes chromosome wide inactivation for the purpose of dosage compensation. The Markov segmentation identifies domains on the X chromosome that correspond to the evolutionary strata that have been proposed earlier [1], [3]. In addition, the major chromosomal changes that have occurred in the X chromosome also leave their distinctive signatures, either by variation in the nucleotide composition or by a characteristic pattern of nucleotide usage, which can be observed as a specific higher–order correlation. The segmentation procedure and the study of selection pressure both suggest that there may be additional structures on the chromosome. The new stratum that we propose could be a consequence of accumulated differences in the sequences *after* the region of DNA was added onto the ancestral mammalian X chromosome. This stratum has not been reported earlier and the present observation raises novel possibilities pertaining to the correlation between gene inactivation and selection pressure.

Genomic regions that are enriched in inactivable genes could probably be more conserved during evolution. The reason, we speculate, is as follows The X chromosome can only recombine in females and therefore X linked genes evolve in a distinctly different manner as compared to the rest of the mammalian genome. Such genes therefore have a differential penetration on the basis of their inactivation status: since only one copy of the inactivated genes is functional in females and there is only one copy of X linked genes in males, any mutation in the inactivable genes would have a greater effect on the phenotype. In light of this hypothesis the genomic region that has a higher number of inactivable genes does tend to show a higher degree of conservation as is demonstrated by our data in this work.

Oligomer frequency analysis of genes that tend to escape inactivation and inactivable domains through TEIRESIAS [14] showed that specific 9– and 10–mers were uniquely present in each class of domain. Although it is unlikely that a single oligonucleotide is responsible for gene silencing, our data suggests that X-inactivation is a “local” process, driven by a small number of nucleotide motifs.

It is plausible that the property of inactivation is spreading from older regions to more recently acquired parts of X. The presence of a single common motif that occurs uniquely in 18 inactivation domains across the X chromosome and the absence of such a consensus in those domains escaping inactivation offers a clue to how such a process could happen. Since this motif occurs only in locations where genes are inactivated, acquisition of this motif might indeed be the crucial step in a new gene gaining the ability to be inactivated. In other words, the unique motifs are likely to be target sites for epigenetic modifications that are needed for the X chromosome inactivation to occur. Modifications like DNA methylation have been shown to be in excess in the inactive X chromosome [20].

Our present results should prove useful for further studies of X chromosomal biology, and especially X inactivation. Markov segmentation provides an accurate estimation of the true domain boundaries: segmentation considers both genes as well as intergenic sequences to identify domains that are homogeneous in composition. It is thus uniquely positioned to give insight into the manner in which sequence composition affects gene inactivation, and can provide a valuable platform for further research into a variety of areas like functional genomics, epigenetics and genome studies in general.

## Supporting Information

Figure S1 Fourth step of segmentation. The JS divergence plot of two intermediate segments (I: 2.88–73.8 Mb, II: 73.8Mb–151.5 Mb) demonstrates prominent peaks at ∼38 Mb and ∼99 Mb, respectively.
(0.13 MB EPS)

Figure S2 Figures A to I show compositional variation in remaining nine domain pairs listed in Table 1.
(2.48 MB EPS)

Table S1 Coordinates of boundaries obtained from six segmentation steps. The data is graphically represented in Figure 1. Segmentation steps having more than one JS divergence maximum are indicated by symbol ‘*’.
(0.05 MB XLS)

Table S2 Synonymous and non-synonymous substitution rates of gene pairs constituted of XAR genes and their orthologs in G. gallus.
(0.11 MB XLS)

Table S3 Expression level of genes from two candidate segments within S3: i) 8.5–38.54 Mb and ii) 38.34–46.5 Mb.
(0.10 MB XLS)

Table S4 Coordinates and gene expression pattern of domain pairs. Genes with normalized expression level >0.75 were labeled transcriptionally ‘Active’, those with < = 0.25 were labeled ‘Inactive’ while those in between of these two were considered having ‘Ambiguous’ expression pattern.
(0.09 MB XLS)

Table S5 Coordinates of domains that are either transcriptionally active or inactive.
(0.05 MB XLS)

## References

[1] Lahn BT, Page DC (1999). Four evolutionary strata on the human X chromosome.. Science.

[2] Lyon MF (1961). Gene action on the X-chromosome of the mouse (Mus musculus L.).. Nature.

[3] Ross MT (2005). The DNA sequence of X-chromosome.. Nature.

[4] Kohn M (2004). Wide genome comparisons reveal the origins of the human X chromosome.. Trends Genet.

[5] Carrel L, Cottle AA, Goglin KC, Willard HF (1999). A first-generation X-inactivation profile of the human X chromosome.. Proc Natl Acad Sci U S A.

[6] Carrel L, Willard HF (2005). X-inactivation profile reveals extensive variability in X-linked gene expression in females.. Nature.

[7] Hall LL, Clemson CM, Byron M, Wydner K, Lawrence JB (2002). Unbalanced X;autosome translocations provide evidence for sequence specificity in the association of XIST RNA with chromatin.. Hum Mol Genet.

[8] Li W, Bernaola-Galvan P, Haghighi F, Grosse I (2002). Applications of recursive segmentation to the analysis of DNA sequences.. Comput Chem.

[9] Azad RK, Lawrence J, Thakur V, Ramaswamy R, Pham TD (2007). Segmentation of genomic DNA sequences.. Advanced Computational Methods in Biocomputing and Bioimaging.

[10] Bernaola Galv'an P, Rom'an Rold'an R, Oliver JL (1996). Compositional segmentation and long-range fractal correlations in DNA sequences.. Phys Rev E.

[11] Lin J (1991). Divergence measures based on the Shannon entropy.. IEEE Trans Inf Theory.

[12] Thakur V, Azad RK, Ramaswamy R (2007). Markov models of segmentation.. Phys Rev E.

[13] Wang Z, Willard HF, Mukherjee S, Fuery TS (2006). Evidence of influence of genomic DNA sequence on human X chromosome inactivation.. PLOS Comp Biol.

[14] Rigoutsos I, Floratos A (1998). Combinatorial Pattern Discovery In Biological Sequences: The TEIRESIAS Algorithm.. Bioinformatics.

[15] Smit AFA, Hubley R, Green P (1996). RepeatMasker Open-3.0.. http://www.repeatmasker.org.

[16] Tatusova TA, Madden TL (1999). Blast 2 sequences - a new tool for comparing protein and nucleotide sequences.. FEMS Microbiol Lett.

[17] Bailey JA, Carrel L, Chakravarti A, Eichler EE (2000). Molecular evidence for a relationship between LINE-1 elements and X chromosome inactivation: the Lyon repeat hypothesis.. Proc Natl Acad Sci U S A.

[18] Carrel L, Park C, Tyekucheva S, Dunn J, Chiaromonte F (2006). Genomic environment predicts expression patterns on the human inactive X chromosome.. PLoS Genet.

[19] Curwen V, Eyras E, Andrews TD, Clarke L, Mongin E (2004). The Ensembl automatic gene annotation system.. Genome Res.

[20] Deobagkar D, Chandra HS (2003). The inactive X chromosome in the human female is enriched in 5-methylcytosine to an unusual degree and appears to contain more of this modified nucleotide than the remainder of the genome.. J Genet.

